# Reversing gp100/IFA-induced impairment of anti-CTLA-4 checkpoint blockade therapy

**DOI:** 10.1186/2051-1426-2-S3-P14

**Published:** 2014-11-06

**Authors:** Yared Hailemichael, Tihui Fu, Hiep Khong, Zhimin Dai, Padmanee Sharma, Willem W Overwijk

**Affiliations:** 1 Department of Melanoma Medical Oncology, The UT MD Anderson Cancer Center, Houston, TX, USA; 2 Department of Immunology, The UT MD Anderson Cancer Center, Houston, TX, USA; 3 Department of Genitourinary Medical Oncology, department of Immunology, The UT MD Anderson Cancer Center, Houston, TX, USA; 4 Department of Melanoma Medical Oncology, The University of Texas Graduate School of Biomedical Sciences at Houston, The UT MD Anderson Cancer Center, Houston, TX, USA

## Background and hypothesis

Cancer immunotherapies have been advanced by the recent FDA approval of anti-CTLA-4 antibody (Ipilimumab, Yervoy^®^) and soon-to-be approved anti-PD-1 antibody, immunological checkpoint-blocking agents with significant anti-tumor activity against melanoma and other cancers. A promising avenue to further increase their efficacy is combination with T cell-inducing vaccination. Surprisingly, addition of gp100 peptide vaccination did not increase but actually decreased clinical efficacy to anti-CTLA-4 in melanoma patients [1]. As a result, it is currently unclear how to combine anti-CTLA-4 with vaccination. We recently reported [2] that vaccination with gp100 peptide in IFA creates a persisting antigen depot that primes antigen-specific CD8+ T cells, followed by their undesirable sequestration at the vaccination site, and eventually their exhaustion and apoptosis, resulting in negligible anti-tumor activity. Here, we investigate whether this phenomenon can also explain the lack of synergy between IFA-based vaccination and anti-CTLA-4 therapy.

## Results

We found that the inflamed, chemokine-rich vaccination site potently attracted and sequestered anti-CTLA-4 activated effector T cells with antigen-specificities unrelated to the gp100/IFA vaccine. Some of the tumor-specific T cells induced by anti-CTLA-4 therapy recognized the melanocyte differentiation antigen, TRP-2, allowing us to quantify their number and localization at the tumor and vaccination site. Anti-CTLA-4 monotherapy significantly increased the absolute number of TRP-2-specific effector T cells at the tumor site at the time of tumor suppression. Remarkably, gp100/IFA vaccination induced sequestration at the vaccination site not only of gp100-specific T cells, but also of TRP-2-specific T cells, dramatically reducing their numbers at the tumor site. In addition, gp100/IFA vaccination slightly reduced therapeutic efficacy of anti-CTLA-4 therapy, replicating the reported clinical observation. In contrast, a non-persistent vaccine formulation, Vesicular Stomatitis Virus encoding gp100 (VSV.gp100) synergized with anti-CTLA-4 to enhance anti-tumor activity. Finally, vaccination also synergized with anti-PD-1 therapy, and with anti-CTLA-4 + anti-PD-1 combination therapy. Immunohistochemistry analysis showed co-localization of CD8+ T cells mainly in ICAM-1 expressing sections of the tumor stroma. ICAM-1 deficiency in host mice resulted in significant abrogation of the anti-tumor activity. Similarly, mice treated with anti-CXCR3 mAb versus control (IgG mAb) showed significant decrease in survival, which correlated with decrease in intra-tumoral CD8+ T cell count at the time of the treatment.

## Conclusion

In conclusion, a non-persistent vaccine formulation can reverse the undesirable effect of the persistent vaccine formulation and synergizes with anti-CTLA-4 and/or anti-PD-1 therapies, resulting in significantly improved anti-tumor activity.
